# Commentary: Treating Pediatric Asthma According Guidelines

**DOI:** 10.3389/fped.2019.00109

**Published:** 2019-03-27

**Authors:** Stanley Szefler, Christian Vogelberg, Branko Jugovic, Alberto de la Hoz, Eckard Hamelmann

**Affiliations:** ^1^The Breathing Institute, Children's Hospital of Colorado and Department of Pediatrics, University of Colorado School of Medicine, Aurora, CO, United States; ^2^University Hospital Carl Gustav Carus, Technical University of Dresden, Dresden, Germany; ^3^TA Respiratory Diseases/Biosimilars Medicine, Boehringer Ingelheim International GmbH, Ingelheim am Rhein, Germany; ^4^Klinik für Kinder- und Jugendmedizin, Evangelisches Klinikum Bethel, Bielefeld, Germany; ^5^Allergy Center of the Ruhr University, Bochum, Germany

**Keywords:** anticholinergics, asthma, lung function, pediatrics, tiotropium

The article by Tesse et al. entitled “Treating Pediatric Asthma According Guidelines” reviewed the conventional and new therapeutic treatment approaches available for pediatric asthma according to guidelines, providing a very good summary of the current pediatric asthma treatment and management landscape from a practical perspective (1). However, the authors incorrectly stated that there were no studies on the use of the anticholinergic agent tiotropium in children with asthma. In this response, we would like to summarize the current indications and available data on tiotropium for those clinicians who might not be aware of the possibility of its use in children.

Several large-scale Phase II and III clinical trials have been conducted to evaluate tiotropium (5 and 2.5 μg doses) delivered via Respimat® as an add-on therapy to at least inhaled corticosteroids (ICS) across a range of asthma severities. These studies involved 1,691 children and adolescents with different asthma severities (Table 1). Based on the efficacy and safety data from the above-mentioned trials, the indication for once-daily tiotropium Respimat® as add-on maintenance therapy for asthma was expanded to include patients aged 6 years and older in the EU (5 μg as two puffs of 2.5 μg once daily) (7) and the USA (2.5 μg as two puffs of 1.25 μg once daily) (8). Indeed, the latest Global Initiative for Asthma recommendations include once-daily tiotropium as a treatment option for addition to ICS plus a long-acting β_2_-agonist at Steps 4 and 5 in adolescents and adults (aged 12 years and over) with a history of exacerbations (9). We briefly summarize below the data published from the clinical studies with tiotropium in children and adolescents.

**Table 1:** Overview of trials with tiotropium Respimat® in adolescents and children with asthma.

**Trial name (NCT number)**	**Persistent asthma severity**	**Baseline therapy**	**Phase**	**Treatment duration, weeks**	**Treatment group, n^**a**^**
**ADOLESCENTS (12–17 YEARS)**					
RubaTinA-asthma® (NCT01257230) (2)	Symptomatic moderate	For 12–14 years: ICS (200–800 μg BUD-eq)^b^ For 15–17 years: medium-dose ICS (400–800 μg BUD-eq)^b^	III	48	Tio 5 μg, 134 Tio 2.5 μg, 125 Placebo, 138
PensieTinA-asthma® (NCT01277523) (3)	Symptomatic severe	For 12–14 years: high-dose ICS (>400 μg BUD-eq) + ≥1 additional controllers^c^ Medium-dose ICS (200–400 μg BUD-eq) + ≥2 additional controllers^d^ For 15–17 years: high-dose ICS (800–1600 μg BUD-eq) + ≥1 additional controllers^c^ Medium-dose ICS (400–800 μg BUD-eq) + ≥2 additional controllers^d^	III	12	Tio 5 μg, 130 Tio 2.5 μg, 127 Placebo, 135
**CHILDREN (6–11 YEARS)**					
CanoTinA-asthma® (NCT01634139) (4)	Symptomatic moderate	Medium-dose ICS (200–400 μg BUD-eq)^b^	III	48	Tio 5 μg, 135 Tio 2.5 μg, 135 Placebo, 131
VivaTinA-asthma® (NCT01634152) (5)	Symptomatic severe	Medium-dose ICS (200–400 μg BUD-eq) + ≥2 additional controllers^d^/high-dose ICS (>400 μg BUD-eq) + ≥1 additional controllers^c^	III	12	Tio 5 μg, 130 Tio 2.5 μg, 136 Placebo, 134
**PRESCHOOL CHILDREN (1–5 YEARS)**					
NinoTinA-asthma® (NCT01634113) (6)	Persistent asthmatic symptoms	Stable ICS dose^b,e^	II/III	12	Tio 5 μg, 31 Tio 2.5 μg, 36 Placebo, 34

*Treated set*.

a *Delivered as two puffs once daily via the Respimat®*.

b *LTRA permitted*.

c *LABA and/or LTRA*.

d *LABA and/or LTRA and/or sustained-release theophylline*.

e *LABA permitted*.

*BUD, budesonide; eq, equivalent; ICS, inhaled corticosteroids; LABA, long-acting β_2_-agonist; LTRA, leukotriene receptor antagonist; NCT, National Clinical Trial; Tio, tiotropium*.

Data have been published from two Phase III studies of tiotropium in adolescents aged 12–17 years. In adolescents with symptomatic moderate asthma, addition of once-daily tiotropium to at least ICS maintenance treatment significantly improved peak forced expiratory volume in 1 second (FEV_1_) within 3 h post-dose (FEV_1(0−3h)_) and trough FEV_1_ (5 μg dose) response, and demonstrated a trend for improved asthma control and quality of life compared with placebo in the RubaTinA-asthma® study (NCT01257230) (2). In the PensieTinA-asthma® study (NCT01277523) involving adolescents with symptomatic severe asthma, although improvements in the primary endpoint—peak FEV_1(0−3h)_ response—were not statistically significant with tiotropium 5 μg vs. placebo, numerical improvements were observed in measures of lung function and asthma control (3).

Two Phase III studies on the efficacy and safety of tiotropium in children aged 6–11 years have also been published. In the CanoTinA-asthma® study (NCT01634139), once-daily tiotropium add-on treatment (both 5 and 2.5 μg doses) significantly increased FEV_1(0−3h)_ and trough FEV_1_ after 24 weeks compared with placebo in children with symptomatic moderate asthma. Both doses of tiotropium also improved asthma control at Weeks 24 and 48 vs. placebo, which were statistically significant with the 5 μg dose at Week 24 (4). In children with symptomatic severe asthma, once-daily 5 μg tiotropium improved lung function outcomes compared with placebo when added to maintenance treatment with ICS plus one or more controller medications in the VivaTinA-asthma® study (NCT01634152) (5).

Finally, in the Phase II/III NinoTinA-asthma® study (NCT01634113), the safety and tolerability of tiotropium was investigated in children aged 1–5 years with persistent asthmatic symptoms despite treatment with at least ICS. Tolerability of tiotropium (both 5 and 2.5 μg doses) was similar to that of placebo, and a *post-hoc* analysis showed both doses of tiotropium reduced adverse events related to asthma worsening compared with placebo (6). However, tiotropium is not currently approved for the treatment of asthma in children below 6 years old.

Tiotropium was well-tolerated across all five trials, with the proportion of patients reporting adverse events and serious adverse events being similar to placebo.

The use of tiotropium in adolescents and children has been discussed in a number of editorial and review articles. For example, in an article by Grigg, he concludes that tiotropium's position in the pediatric management pathway remains unclear, citing heterogeneous data from the adolescent studies RubaTinA-asthma and PensieTinA-asthma (10). Other articles present a different opinion, citing the pooled data from all the published trials in pediatric patients that provide evidence and support for the efficacy of tiotropium in adolescents and children (11–14). However, we agree there is a need for more studies in children and adolescents to compare step-up treatment options and provide an evidence-based stepwise approach for the management of asthma in children (15).

In conclusion, there is a comprehensive published clinical trial program with tiotropium in children and adolescents with different severities of asthma that has led to approval for its use in children aged from 6 years in the EU and the USA.

## Author Contributions

SS, CV, and EH were involved in the clinical studies and publications cited in the commentary. BJ and AdlH were involved in the conceptual development of the commentary.

### Conflict of Interest Statement

SS has received payments to his institution from Aerocrine, AstraZeneca, Boehringer Ingelheim, Daiichi Sankyo, GlaxoSmithKline, Genentech, Novartis, Propeller Health, Regeneron and Sanofi, and has received research support from GlaxoSmithKline. CV has consulted for Allergopharma, ALK, Bencard, Boehringer Ingelheim, Novartis, Stallergenes, Sanofi Avensis, Engelhard, DBV Technology and has received research support from the German Society of Research (DFG). BJ and AdlH are employees of Boehringer Ingelheim. EH has consulted for Allergopharma, ALK, Bencard, Boehringer Ingelheim, GlaxoSmithKline, HAL Allergy, Novartis, Nutricia and Stallergenes, and has received research support from the German Society of Research (DFG), the State Ministry of Education and Research of Nordrhein-Westfalen (LMBF), and the German Ministry of Education and Research (BMBF).
